# Mechanical Properties of Basalt-Fiber-Reinforced Metakaolin–Slag–Fly Ash Geopolymer Mortar Characterized by 2D-DIC

**DOI:** 10.3390/ma19132729

**Published:** 2026-06-25

**Authors:** Renfei Gao, Lianyong Zhu, Pengchang Liang, Weizi Wang, Ruize Yin

**Affiliations:** College of Water Resources and Architectural Engineering, Tarim University, Alar 843300, China; 15588733729@163.com (R.G.); 10757232189@taru.edu.cn (P.L.); wangweizi1224@163.com (W.W.); 10757232192@taru.edu.cn (R.Y.)

**Keywords:** basalt fiber, geopolymer mortar, two-dimensional digital image correlation (2D-DIC), reinforcement mechanism

## Abstract

Against the backdrop of rapid development in low-carbon building materials, geopolymer mortar has become a high-quality alternative to traditional cement-based materials due to its advantages of environmental friendliness, high strength, and excellent durability. However, its inherent brittleness and tendency to crack severely limit its widespread adoption and use in engineering. To mitigate the inherent brittleness of geopolymer mortar, this study developed a ternary binder system composed of metakaolin, slag, and fly ash. The effects of basalt fiber contents of 0%, 0.25%, 0.50%, 0.75%, 1.00%, and 1.25% by mass on the flowability, flexural strength, compressive strength, and deformation behavior of the geopolymer mortar were systematically investigated. The evolution of the displacement and strain fields during flexural and compressive loading was monitored in real time using two-dimensional digital image correlation (2D-DIC). The fiber-reinforcement mechanism was further examined by X-ray diffraction (XRD), scanning electron microscopy (SEM), and Fourier transform infrared spectroscopy (FTIR). The results show that basalt fiber reduces mortar flowability, and the reduction becomes more pronounced with increasing fiber content. The flexural strength first increased and then decreased with increasing fiber content; at 0.50% fiber content, the 28-day flexural strength reached 12.6 MPa, which was 8.2% higher than that of the fiber-free control. The compressive strength increased only slightly at a low fiber content of 0.25% and then decreased when the fiber content exceeded 0.50%. The 2D-DIC results indicate that a moderate fiber content (0.50–0.75%) markedly increased the ultimate displacement, delayed crack propagation, and enhanced the post-cracking deformation capacity. Microstructural observations revealed that an appropriate fiber content promoted good interfacial bonding with the matrix and enabled fiber bridging and crack resistance. In contrast, excessive fiber addition caused agglomeration-induced micropores and microcracks, thereby degrading mechanical properties. Overall, the recommended basalt fiber content is 0.25–0.50%. These findings provide a theoretical and experimental basis for optimizing high-performance, low-carbon geopolymer mortar for engineering applications.

## 1. Introduction

The manufacture of ordinary Portland cement is characterized by intensive energy consumption and considerable CO_2_ emissions. Alongside the rapid advancement of urbanization and expansion of global infrastructure, this drawback has become a critical bottleneck hindering sustainable development [1]. Global anthropogenic CO_2_ emissions see roughly 7–8% contributed by the cement industry [2]. Accordingly, the development of environmentally friendly and high-performance cementitious materials as partial or complete substitutes for conventional cement has become an important research focus in civil engineering materials [3,4,5].

Geopolymers are three-dimensional inorganic polymers produced from aluminosilicate precursors, such as metakaolin and industrial by-products, through alkali-activated depolymerization and polycondensation. Owing to their relatively low carbon footprint, excellent high-temperature resistance, good acid resistance, and strong capacity for heavy-metal immobilization, geopolymers are widely regarded as promising alternatives to Portland cement [6,7,8]. Kumble et al. reported that alkali-activated building bricks bonded with alkali-activated mortar possessed greater bond strength compared with bricks bonded by traditional cement mortar [9]. Other studies have shown that alkali-activated slag mortar can offer better durability than ordinary Portland cement mortar under certain aggressive environments [10]. Cheng et al. further reported that geopolymer mortar exhibits superior strength and corrosion resistance compared with commonly used OPC mortar, indicating its potential for highly erosive engineering environments [11].

However, the high brittleness and tendency toward shrinkage-induced cracking of geopolymer materials severely restrict their large-scale use in load-bearing structural members and harsh service environments [12,13]. This limitation is mainly related to the rigid inorganic network of geopolymer gels, including N-A-S-H and C-(N)-A-S-H, which have relatively low fracture toughness. Under tensile or flexural loading, these materials are therefore prone to brittle failure, and once cracks initiate, they can propagate rapidly [14]. Improving the toughness of geopolymers and suppressing the initiation and growth of microcracks remain key scientific challenges for their transition from laboratory research to practical engineering applications. Clarifying their fracture behavior and improving their resistance to brittle failure are therefore essential for ensuring structural safety and service reliability.

Inspired by toughening strategies for conventional cement-based materials, the incorporation of short chopped fibers into brittle matrices is widely considered one of the most effective and economical approaches [15,16,17]. Polypropylene, polyvinyl alcohol, steel, and plant fibers have been used to reinforce geopolymers, leading to different degrees of improvement in strength and toughness [18,19,20,21]. Kozub et al. prepared fly ash-sourced geopolymer composite materials reinforced with diverse contents of melamine fibers and found that an appropriate fiber content effectively improved compressive strength [22]. Lin et al. reported that wood fibers reduced the brittleness of silicoaluminophosphate geopolymers and improved fracture toughness, with good bonding between the fibers and reaction products [23]. Li et al. reported that the fracture toughness of polypropylene fiber-reinforced geopolymers first increased and then decreased with the reinforcement index, and that polypropylene fibers provided a more pronounced toughening effect and better cost efficiency than other fiber types [18]. Among these reinforcements, basalt fiber (BF), a green inorganic mineral fiber, has drawn growing interest owing to its superior specific strength and elastic modulus, high-temperature and corrosion resistance, and good compatibility with aluminosilicate matrices due to its SiO_2_- and Al_2_O_3_-rich composition [24,25,26,27]. Compared with organic synthetic fibers, basalt fiber is more environmentally friendly and provides better long-term stability; compared with steel fiber, it has lower density and higher corrosion resistance, thereby avoiding rust-related durability problems [28,29]. Specific research has verified relevant findings by Gabriel Furtos et al.: incorporating MiniBars™ at volume fractions of 0.5–75% yields dramatic performance enhancements for modified composites in comparison with the plain unreinforced geopolymer matrix. To be specific, bending strength improves by a factor ranging from 11.59 to 25.97, flexural modulus sees a 3.33–5.92-fold increment, while tensile strength gains 3.50–8.03 times, tensile modulus by 1.12–1.30 times, and upper yield strength load by 4.18–7.27 times [30]. In another study conducted by Limin Lu et al., the inclusion of basalt fiber leads to notable improvements in the thermal insulation properties of concrete and alleviates concrete spalling [31]. These characteristics make basalt fiber a suitable reinforcement for geopolymer composites.

As a non-destructive, whole-field optical deformation measurement technique, digital image correlation (DIC) tracks grayscale changes in speckle patterns on specimen surfaces to deliver high-precision displacement and strain fields under loading conditions [32,33,34]. Applying DIC to monitor the bending and compressive fracture behaviors of basalt fiber-toughened geopolymer mortar can help bridge the gap between macroscopic mechanical responses and microscopic damage mechanisms. Liu et al. investigated carbon fiber-reinforced coral concrete using four-point bending tests combined with DIC and found that the damage process consisted of three stages. Carbon fibers prolonged deformation, improved flexural performance, and transformed the failure pattern from brittle failure to ductile behavior; at an optimal content of 1.5%, bending strength rose 20.8–33.3%, peak displacement climbed 116.2–142.1%, and toughness surged by 367–586%, respectively [35]. Enfedaque et al. used fracture energy tests and DIC to show that incorporating 25% volcanic ash or thermally treated kaolin significantly increased the fracture energy of glass fiber-reinforced cement (GRC), while also confirming the applicability of DIC to GRC fracture testing [36]. Huang et al. used DIC to study concrete with different water-to-cement ratios and observed uneven axial displacement distributions, with larger displacements at the matrix-fiber interface and mortar than in the aggregates. Deformation localization appeared at 30–40% of peak stress, and higher water-to-cement ratios produced more and larger localized zones. Microcracks nucleated within the fiber–matrix interfacial region, and an elevated water-binder ratio triggered more widespread cracking across broader areas, accompanied by reduced crack apertures [37].

Accordingly, a ternary cementitious system consisting of metakaolin, slag, and fly ash was adopted in this study to fabricate geopolymer mortar with basalt fiber mass dosages of 0, 0.25%, 0.50%, 0.75%, 1.00%, and 1.25%, and its mechanical properties and deformation behaviors were systematically investigated. First, macro-mechanical indexes were acquired via flexural and compressive tests to clarify their variation laws with fiber dosage so as to determine the optimal range of fiber content. On this basis, the in situ two-dimensional digital image correlation (2D-DIC) technique was applied innovatively to monitor in real time the evolution of surface displacement and strain fields of specimens under loading. The strain localization process, crack initiation, and propagation characteristics at different fiber dosages were quantitatively analyzed, revealing the restraint and bridging effects of fibers on the matrix. Finally, multiple microscopic characterization techniques, including X-ray diffraction (XRD), scanning electron microscopy (SEM), and Fourier-transform infrared spectroscopy (FTIR), were comprehensively utilized. The intrinsic strengthening and toughening mechanisms of basalt fiber for geopolymer mortar were deeply elaborated from multiple perspectives such as phase composition, micromorphology, interfacial structure and chemical bonding state. The outcomes of this research are expected to provide systematic theoretical foundations and experimental data for the compositional optimization design of basalt-fiber-reinforced geopolymer mortar, and promote technological progress and engineering application of high-performance, low-carbon green building materials.

## 2. Experimental Program

### 2.1. Raw Materials

The metakaolin-slag-fly ash-based geopolymer mortar (MSF-GPM) prepared in this study consisted of metakaolin, ground granulated blast-furnace slag, fly ash, sand, an alkaline activator, and basalt fibers. The geopolymer binder was a ternary composite consisting of metakaolin, slag, and fly ash, and its chemical composition is listed in Table 1. Metakaolin, sourced from Chenyi Refractory Abrasive Co., Ltd. (Gongyi, Henan, China), exhibited strong pozzolanic activity, featuring 1250 mesh fineness and an activity index of 110; its SiO_2_ and Al_2_O_3_ contents were 52.22% and 43.49%, respectively. The abundant silicon and aluminum oxides provide essential aluminosilicate precursors, which are the fundamental raw materials for forming the N-A-S-H geopolymer gel network under alkaline activation. The slag was grade S95 slag supplied by Bairun Refractory Material Co., Ltd. (Gongyi, Henan, China). The material was obtained through furnace quenching, drying, and grinding and showed potential hydraulic reactivity; its SiO_2_ and Al_2_O_3_ contents were 31.44% and 14.29%, respectively. Rich calcium oxide in slag introduces a calcium source into the system, facilitating the generation of C-(N)-A-S-H mixed gel and improving matrix compactness via synergistic hydration and geopolymerization. Fly ash was supplied by Guizhou Huasheng Testing Co. (Anshun, Guizhou, China), Ltd. It had a fineness of 8.2%, a water demand ratio of 93%, and a loss on ignition of 4.13%; its SiO_2_ and Al_2_O_3_ contents were 69.35% and 18.53%, respectively. The high silica content of fly ash further supplements silicate components, optimizing the overall Si/Al ratio of the ternary binder and guaranteeing sufficient progress in alkali-activated polymerization.

Raw materials for fabricating the alkali activator in the present work included water glass (sodium silicate solution), pelletized sodium hydroxide, and mixing water. The sodium silicate solution, sourced from Zhichen Refractory Material Co., Ltd. (Jiaxing, Zhejiang, China), possessed an initial modulus of 2.3, expressed as the molar proportion of SiO_2_ relative to Na_2_O. Pellet sodium hydroxide with 96% purity, obtained from Puhui Chemical Raw Materials & Reagents Co., Ltd. (Shaoguan, Guangdong, China), was adopted to tune the silicate modulus of the final activator to 1.5. Table 2 presents the chemical indices of the sodium silicate solution. River sand from natural sources, possessing a fineness modulus of 2.53 and apparent density reaching 2635 kg/m^3^, acted as fine aggregate within the geopolymer matrix. Surface-modified basalt fiber via silane coupling agent was chosen as the reinforcement filler, which was provided by Changsha Ningxiang Building Materials Co., Ltd. (Changsha, Hunan, China). These basalt fiber filaments measured 6 mm in length, with a bulk density of 2.62 g/cm^3^, tensile strength of 1938 MPa, and elastic modulus of 76.1 GPa, average diameter around 17 μm, and breaking elongation of 2.9%, demonstrating outstanding mechanical strength and rigidity. Table 3 presents all key performance indicators of adopted raw materials.

### 2.2. Mix Design

In accordance with preliminary trials and published studies [38], the silicate modulus of the alkaline activator was set to 1.5, the alkali equivalent was kept at 8%, while water-binder and sand-binder ratios were maintained at 0.45 and 3:1, respectively. The binder consisted of metakaolin and slag at a mass ratio of 5:5, with 10% fly ash added externally. With this benchmark mixing ratio adopted, the basalt fiber dosage was taken as the sole variable, with mass fractions of 0%, 0.25%, 0.50%, 0.75%, 1.00%, and 1.25% relative to the total weight of the geopolymer mortar. The detailed mix proportions are given in Table 4.

### 2.3. Specimen Preparation

**Figure 1:**
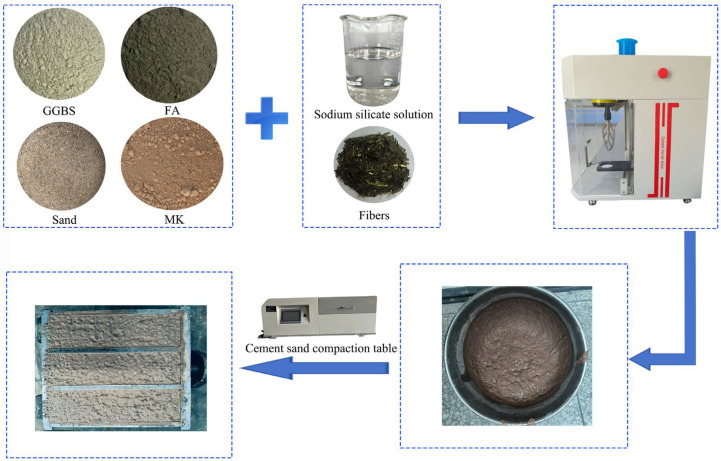
Flow chart of mortar preparation.

Figure 1 illustrates the specimen preparation process adopted in this test. First, the alkali activator was prepared according to the designed mix proportion: solid sodium hydroxide was dissolved in sodium silicate solution, and the mixed solution was kept standing at room temperature for 24 h before application. In particular, a set dosage of water glass solution was poured into a mixing container; solid sodium hydroxide was gradually incorporated while stirring constantly to achieve full dissolution, which adjusted the modulus of the alkali activator to the target test value. All raw materials and mixing water were accurately weighed for standby use strictly in accordance with the predetermined mix proportion. For specimen mixing, the binder was initially added to the mixer and subjected to dry mixing over a 30 s period. The cooled alkali activator was then added and mixed for another 30 s. Standard sand was blended evenly into the mixture within 30 s, then high-speed mixing was performed for another 30 s. Following a 90 s stationary interval after mixer shutdown, residual mortar attached to mixing components and bowl inner surfaces was collected at the bowl bottom. Subsequent high-speed mixing lasting 60 s finalized the full mixing sequence of samples [39].

Immediately after mixing, the mortar was filled into the mold cavities. Empty molds (40 mm × 40 mm × 160 mm) and the mold frame were fixed on a vibrating table. The mortar adhering to the bowl wall was scraped off and mixed thoroughly. The mortar was then placed into the molds in two layers. For the first layer, approximately 300 g of mortar was added to each compartment. A longitudinal slicing motion was used to fill the compartment completely, followed by leveling with a large spreader and 60 jolts. After the second layer was added, the mortar was again sliced and spread to fill the mold without disturbing the first layer. A smaller spreader was used for leveling, followed by another 60 jolts. During vibration, a wet cotton cloth could be placed over the mold to prevent splashing. The mold frame was then removed, and the molds were taken off the vibrating table. A metal straightedge was placed at the end of the mold at an angle close to 90° (slightly inclined toward the scraping direction) and used to remove excess mortar longitudinally. The number of sawing strokes and the scraping angle were adjusted according to mortar consistency; for stiffer mortars, more strokes and slower movements were required to avoid disturbing the compacted portion. A wrung-out wet towel was used to clean the mortar from the top of the mold end plates. The same straightedge was then used to smooth the specimen surface at an almost horizontal angle, with as few passes as possible (no more than three). Finally, the mortar around the mold was cleaned, and the specimens were labeled. After standard curing for 24 h, the specimens were demolded and transferred to a standard curing room (20 ± 1 °C, relative humidity > 90%) until the designated testing ages [39].

### 2.4. Test Methods

#### 2.4.1. Strength Test

Strength tests were conducted in accordance with GB/T 17671-2021 [39], Test Method for Cement Mortar Strength (ISO Method). After curing to the specified age, the specimens were removed from the curing room. Flexural tests were performed first, and the fractured half-prisms were then immediately used for compressive tests [39]. Flexural tests were conducted first on prism specimens measuring 40 mm × 40 mm × 160 mm. Each flexural specimen broke into two half-prisms, which were then immediately used for compressive tests. For the compressive test, the specimen was placed with its side face between the platens, and a continuous and uniform load was applied at 2400 N/s until failure. The ultimate breaking load was documented. The calculation formula is as follows:

(1) $$R_{c}\;=\;\frac{F_{c}}{A}$$

where

$R_{c}$ is the compressive strength (MPa);
$F_{c}$ is the maximum failure load (N);
*A* is the compression area (mm^2^) [39].

Samples were stripped from molds after 24 h of sealed curing, then maintained until the designated test age. Flexural strength measurement complied with Chinese specification GB/T 17671-2021 [39], also named the Test Method for Cement Mortar Strength (ISO Procedure). In this study, all test specimens were prepared with a dimension of 40 mm × 40 mm × 160 mm, and three parallel specimens were adopted for each group to ensure data reliability. During testing, each specimen was placed with its side face on the support rollers, with its longitudinal axis perpendicular to the rollers. A vertical load was applied through the loading roller at 50 N/s until fracture, and the maximum load at failure was recorded. The calculation formula is as follows:

(2) $$R_{f}=\;\frac{1.5F_{f}L}{b^{3}}$$

where

$R_{f}\;$ is the flexural strength (MPa);
$F_{f}\;$ is the load applied at the midspan of the prism at fracture (N);
*L* is the distance between the support rollers (mm);
*b* is the side length of the square cross-section of the prism (mm) [39].

The final test results were averaged from valid measured data. Outliers deviating greatly from the average were eliminated in accordance with relevant specifications to ensure the accuracy and repeatability of experimental data. All groups of data show favorable stability and low dispersion, and the test results possess sound statistical validity and reproducibility.

#### 2.4.2. Flowability Test

Mortar fluidity was measured referring to the national standard GB/T 2419-2005 [40], Test Method for Fluidity of Cement Mortar. If the flow table had been idle for more than 24 h, pre-operation with one cycle of 25 unloaded jolts was required prior to formal testing. Before conducting measurements, the surfaces of the flow table and interior walls of the truncated conical mold, tamper, and all tools in contact with mortar were wiped with a damp cotton cloth. Next, the mold was arranged at the flow table’s center and wrapped with damp fabric for standby application. The detailed testing procedures are described as follows: Fresh mortar was rapidly filled into the truncated cone mold on the flow table in two layers. The initial layer, which filled approximately two-thirds of the mold’s height, was cut five times along two perpendicular directions with a knife, and was compacted uniformly 15 times from the periphery to the center. The second layer was filled until the mortar was approximately 20 mm higher than the top edge of the mold. After repeating five perpendicular cuts, 10 uniform tamping strokes were applied from the edge toward the center. Upon finishing compaction, the mold sleeve was removed. A knife was held nearly horizontally to trim excess mortar above the mold in two strokes from the center outward, and any spilled mortar on the table was wiped off. The truncated cone mold was lifted slowly and vertically until fully separated from the mortar pile. The flow table was activated immediately to deliver one jolt per second, finishing 25 jolts within 25 ± 1 s. After jolting, calipers were used to measure the spread diameters of the mortar cake along two perpendicular orientations. The average diameter value, rounded to the nearest millimeter, was defined as the mortar fluidity [40]. Several key precautions were followed throughout the test: the mortar surface after tamping should slightly exceed the mold top; the first layer was compacted down to half its original height, and the compacted second layer sat marginally above the consolidated first layer; the mold was held firmly during filling and tamping to prevent displacement; the entire fluidity test, from the start of water mixing to the completion of diameter measurement, had to be accomplished within 6 min.

#### 2.4.3. DIC Test

The DIC measurement system consisted of a camera, an optical lens, an image acquisition card, a computer, and a storage medium [41] and enabled the complete workflow for speckle-image acquisition, storage, analysis, and calculation [42]. A high-contrast random speckle pattern was first prepared on the test surface of each specimen. During testing, the loading machine applied load while the computer software simultaneously triggered the camera to continuously capture speckle images of the specimen surface at a preset frequency and save them in real time. The collected image sequence was subsequently post-processed using XTDIC (version 9.7.2) professional DIC analysis software. By tracking the grayscale distribution of speckle subsets before and after deformation, the software calculated the two-dimensional displacement field at each point on the specimen surface using a correlation algorithm. The full-field strain, including normal strain and shear strain components, was then obtained through numerical differentiation. Analysis of strain localization and its evolution enabled accurate identification of crack initiation sites, tracing of crack propagation paths, and quantitative characterization of the damage development process.

#### 2.4.4. SEM Test

Field-emission scanning electron microscopy with high resolution (SEM) was used to observe the specimen microstructure and fiber–matrix interfacial bonding. For sample preparation, specimens cured for 28 days were mechanically fractured, and fresh fragments were taken from the central region of the fracture surface while avoiding carbonated or surface-contaminated areas, thereby ensuring representative and undisturbed microstructural observations. To stop further hydration of residual binder components, the selected fragments were submerged in absolute ethanol with a 48 h soaking period. They were then dried in a constant-temperature oven at 60 °C to constant mass. A thin gold film was sputtered onto the sample surface using an ion sputter coater (Quorum Technologies Limited, Laughton, UK) to enhance secondary-electron emission and eliminate charging effects during high-magnification imaging. Observations were carried out using a Sigma 300 high-resolution field-emission SEM (ZEISS, Oberkochen, Germany) at an appropriate accelerating voltage to analyze the relationship between microstructural characteristics and the macroscopic mechanical and durability performance of the material.

#### 2.4.5. XRD Test

An X-ray diffractometer (XRD) was used to analyze the phase composition and crystal-structure evolution of the reaction products. To ensure representative and accurate results, fresh samples were drilled from the central region of specimens with each mix proportion before testing. After removal of carbonated or surface-contaminated portions, the samples were dried in a constant-temperature oven at 60 °C to constant mass. The dried samples were ground in an agate mortar until they passed through a 200-mesh standard sieve, which reduced the effect of particle orientation on diffraction intensity and yielded well-dispersed powder samples. XRD measurements were performed using a Rigaku SmartLab SE X-ray diffractometer (Rigaku, Akishima-shi, Japan) with copper (Cu) target radiation. The scanning range was set as 10–80° (2θ), with a scanning speed of 10°/min and a step size of 0.02°. Phase identification and semi-quantitative analysis based on the position, intensity, and shape of characteristic peaks were used to reveal the reaction degree, product composition, and their influence on the macroscopic performance of the geopolymer system at different mix proportions.

#### 2.4.6. FTIR Test

Fourier-transform infrared spectroscopy (FTIR) was used to analyze the functional groups and chemical-structure evolution of the reaction products under different mix proportions and curing conditions, and to further examine the molecular-structure evolution and chemical bonding mechanism of the gel phase during geopolymerization. For sample preparation, specimens cured for 28 days were mechanically fractured, and fresh particles from the central region were selected. The samples were immersed in anhydrous ethanol for 48 h and then dried in a constant-temperature oven at 60 °C to constant mass. They were subsequently ground in an agate mortar to a particle size below 5 μm to reduce the influence of particle scattering on the infrared baseline. A conventional powder-pellet method was used for testing. Spectra were collected using a Nicolet iS20 Fourier transform infrared spectrometer (Thermo Scientific, Madison, WI, USA) over a wavenumber range of 4000–400 cm^−1^, with a resolution of 4 cm^−1^ and 32 scans. Shifts, intensity variations, and shape changes in characteristic absorption bands, such as Si-O-T bonds and hydration products, were analyzed to clarify the chemical bonding state of the geopolymer gel phase under different mix proportions and its relationship with macroscopic performance.

## 3. Results and Discussion

### 3.1. Flowability

**Figure 2:**
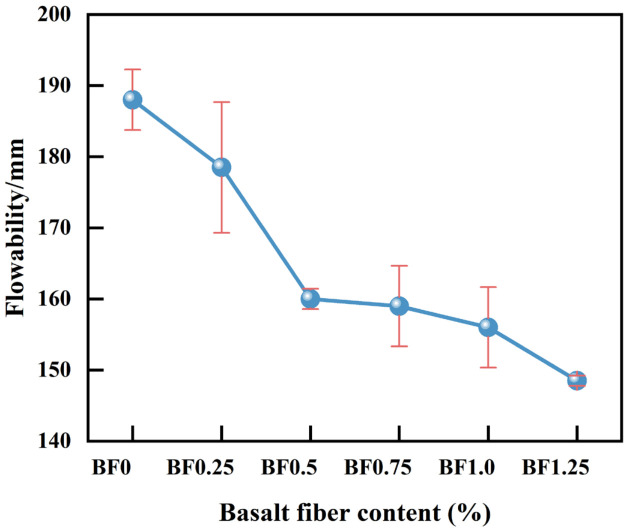
Flowability of geopolymer mortar.

Figure 2 presents the flowability results of geopolymer mortar with different basalt fiber contents. Basalt fiber content had a clear influence on mortar workability. The flowability of the control group (BF0) was 188 mm. As fiber content increased, flowability decreased continuously: 178.5 mm at 0.25% (BF0.25), 160 mm at 0.50% (BF0.5), 159 mm at 0.75% (BF0.75), 156 mm at 1.00% (BF1.0), and 148.5 mm at 1.25% (BF1.25). Compared with the control, the corresponding reductions were 5.1%, 14.9%, 15.4%, 17.0%, and 21.0%, respectively. Thus, basalt fiber incorporation significantly reduced geopolymer mortar flowability, and the reduction became more pronounced as fiber content increased. This decrease can mainly be attributed to the high specific surface area of basalt fibers, which adsorbs part of the free water and paste during mixing. In addition, physical cross-linking and entanglement among fibers increase internal frictional resistance within the paste [43,44]. The monotonic declining trend of flowability with rising basalt fiber dosage obtained in this research is consistent with the universal conclusion of existing relevant research [45].

### 3.2. Flexural Strength

**Figure 3:**
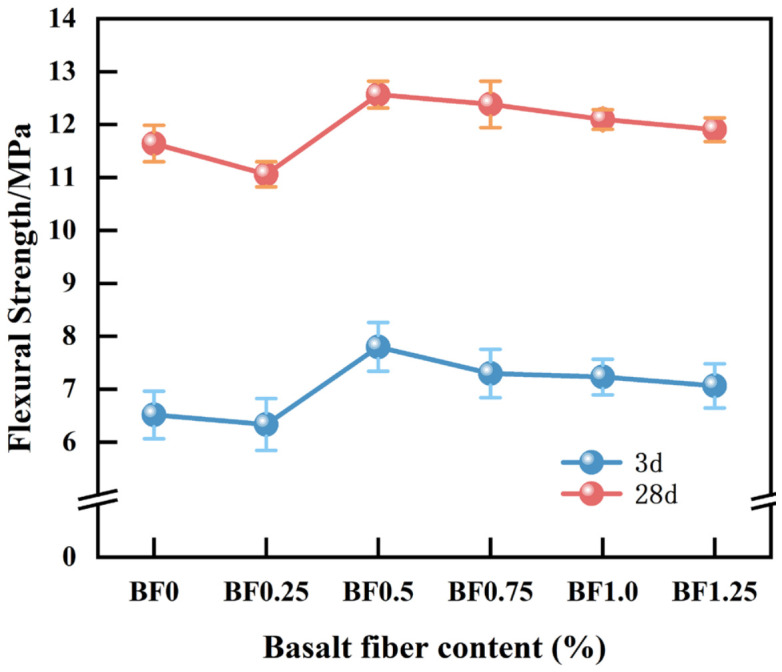
Flexural strength.

Figure 3 shows the flexural strength of geopolymer mortar at different ages with various basalt fiber contents. The fiber-free control (BF0) had 3-day and 28-day flexural strengths of 6.51 MPa and 11.64 MPa, respectively. As fiber content increased, the 3-day flexural strength first decreased slightly to 6.33 MPa for BF0.25 and then increased to a maximum of 7.80 MPa for BF0.5, corresponding to an increase of approximately 19.8% relative to the control. With further increases in fiber content (BF0.75, BF1.0, and BF1.25), the 3-day flexural strength decreased but remained higher than that of the control. For 28-day flexural strength, BF0.25 decreased to 11.1 MPa, a reduction of approximately 4.9%; BF0.5 reached a maximum of 12.6 MPa, 8.2% higher than the control; thereafter, BF0.75, BF1.0, and BF1.25 showed a gradual decline but remained close to the control value. These results indicate that 0.50% basalt fiber effectively improved flexural strength, particularly at an early age, whereas excessive fiber content (1.00% or higher) weakened the reinforcing effect [46]. At very low fiber content, the fibers cannot form an effective three-dimensional network in the matrix, and the number of fibers per unit volume is insufficient to provide pronounced bridging and crack-resisting effects. The fiber–matrix interfacial transition zone may instead introduce local weak points, which may explain why BF0.25 showed lower flexural strength than BF0 at both 3 and 28 days [47].

The obvious discrepancy of reinforcing efficiency between early and late curing ages originates from the continuous development of the geopolymer gel microstructure. At the early curing stage of 3 d, incomplete geopolymerization leaves abundant inherent microcracks within the ternary binder matrix; uniformly dispersed basalt fiber at a 0.5% dosage builds a continuous crack-bridging network to restrain crack expansion under bending load, hence bringing a prominent enhancement in early flexural performance. After 28 d of curing, sufficient formation of N-A-S-H and C-(N)-A-S-H gels densifies the original matrix and eliminates most initial micro-defects, which reduces the improvement potential of the fiber [48]. The optimal basalt fiber content of approximately 0.5% for flexural performance obtained in this study falls within the reasonable range of 0.2% to 0.6% reported in the existing literature [49].

### 3.3. Compressive Strength

**Figure 4:**
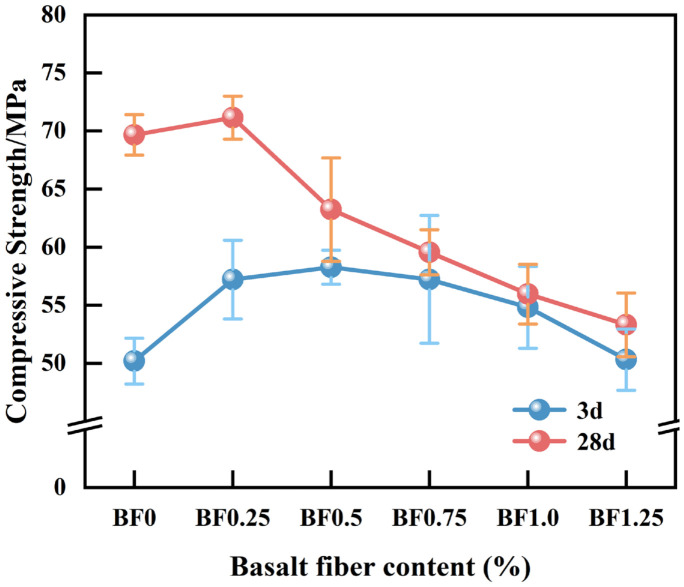
Compressive strength.

Figure 4 shows the compressive strength of geopolymer mortar at different ages with various basalt fiber contents. At all ages, strength generally increased at low fiber contents and decreased at high fiber contents. The 3-day and 28-day compressive strengths of the control group were 50.2 MPa and 69.85 MPa, respectively. At low fiber contents of 0.25% and 0.50%, the 3-day compressive strengths increased markedly to 57.21 MPa and 58.27 MPa, corresponding to increases of 14–16%. However, at 0.50% fiber content, the 28-day strength decreased to 63.23 MPa, a reduction of 9%; at 0.75%, it further decreased to 59.56 MPa, a reduction of 15%. At 1.00% fiber content, the 28-day compressive strength was 55.95 MPa, a decrease of 20%, and at 1.25%, the 3-day strength remained almost unchanged at 50.32 MPa, whereas the 28-day strength decreased sharply to 53.30 MPa, a reduction of 24%. An appropriate amount of basalt fiber can enhance early-age compressive strength because the fibers bridge and resist microcracks, delay crack propagation, improve structural integrity, and contribute to stress transfer and energy dissipation [46]. At 28 days, however, only the very low fiber content retained a slight strengthening effect, while contents of 0.50% and above produced strengths lower than that of the fiber-free control. Once the fiber content exceeds a critical threshold, the strength contribution weakens or becomes negative because excess fibers tend to agglomerate and disperse unevenly, forming local weak interfaces and pore defects that impair mechanical performance [50].

The obvious difference in reinforcing efficiency of basalt fiber between early and long curing ages is closely correlated with the evolution degree of geopolymer gel. At the early curing stage, incomplete geopolymerization leaves abundant inherent microcracks inside the matrix. Well-dispersed basalt fibers effectively restrain crack propagation under external compression and deliver prominent reinforcing performance. As the alkali-activated reaction proceeds continuously up to 28 days, abundant N-A-S-H/C-(N)-A-S-H gels fill internal pores and densify the matrix, which weakens the improvement effect of low-dosage fibers on inherent defects [51]. Excessive fiber incorporation introduces extra fiber–matrix interfacial gaps and agglomeration-induced voids, which gradually develop into critical damage sources under compressive loading and markedly degrade the late-age compressive strength of mortar [50].

The optimal basalt fiber content of approximately 0.25% for improving flexural performance in this study is within the optimal range of 0.2% to 0.6% reported in the existing literature [49].

### 3.4. DIC-Based Analysis of Flexural Failure Evolution

#### 3.4.1. Characteristic-Point Displacement and Deformation Capacity

**Figure 5:**
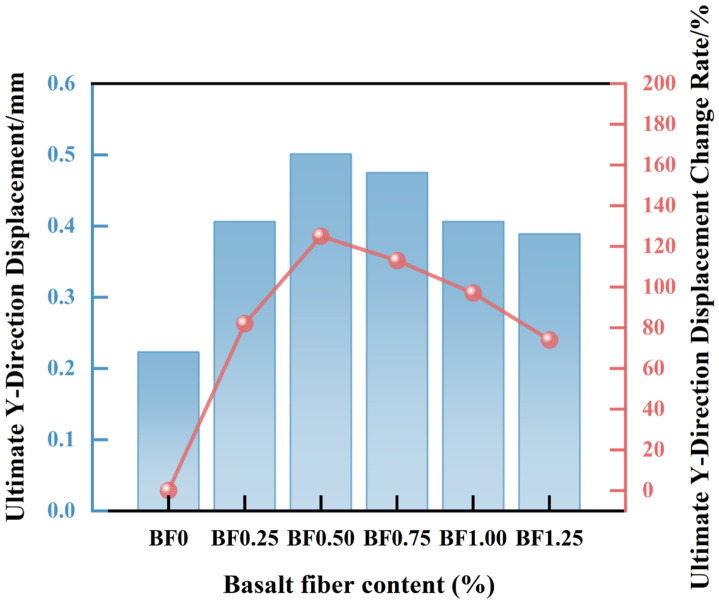
Ultimate displacement in the Y direction.

Based on the Y-direction displacement data of characteristic points recorded during the 28-day flexural tests, the maximum displacement before failure was extracted as a quantitative indicator of deformation capacity for specimens with different fiber contents. Using six sets of in situ 2D-DIC displacement-monitoring data, the effects of basalt fiber contents of 0%, 0.25%, 0.50%, 0.75%, 1.00%, and 1.25% on the deformation characteristics of geopolymer mortar were systematically analyzed. The ultimate displacements of specimens with different fiber contents are shown in Figure 5. The ultimate displacement of the fiber-free specimen was only 0.223 mm, indicating rapid failure after peak load and typical brittle behavior. After basalt fiber incorporation, the ultimate displacement increased markedly. The 0.50% and 0.75% groups reached ultimate displacements of 0.501 mm and 0.475 mm, respectively, which were 125% and 113% higher than that of the fiber-free group. This clearly demonstrates the toughening effect of basalt fibers, which improved post-cracking deformation capacity by bridging cracks and delaying crack propagation [52]. The ultimate displacements of the 0.25% and 1.00% groups were 0.406 mm and 0.439 mm, respectively. Overall, a moderate fiber content substantially improved deformation capacity. When the fiber content increased to 1.25%, the ultimate displacement decreased to 0.389 mm. Although still higher than that of the fiber-free group, it was lower than that of the 0.50% group, indicating that local interfacial weakening caused by fiber agglomeration began to adversely affect ductility.

The incorporation of basalt fiber significantly improved the deformation capacity of geopolymer mortar. The ultimate displacement in the Y direction first increased and then decreased with increasing fiber content. When an appropriate amount of basalt fiber was uniformly dispersed in the matrix, the fiber-bridging effect effectively inhibited crack propagation, delayed unstable failure, and transformed the failure mode from brittle fracture toward ductile failure, showing a clear toughening effect. At excessive fiber contents, agglomeration introduced more local interfacial defects, reduced fiber–matrix cooperation, and weakened the reinforcement effect.

#### 3.4.2. Transverse Deformation at the Crack Location

**Figure 6:**
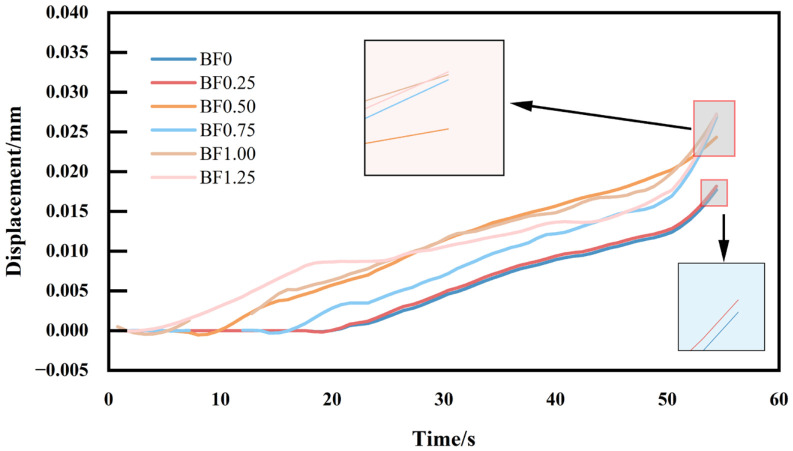
Transverse deformation at the crack location.

Figure 6 shows the transverse deformation at the crack location for specimens with different fiber contents. The evolution of transverse deformation differed considerably among the groups. In the initial deformation stage, only BF1.25 (1.25% fiber content) exhibited positive deformation, whereas the other groups showed no positive deformation at this stage. As loading continued, BF0.5 (0.50% fiber content) began to show positive deformation at 12 s, while BF0 and BF0.25 began to show positive deformation at approximately 21 s. The specimen with the highest flexural strength (BF0.5) did not exhibit the largest final positive transverse deformation, indicating that this fiber content produced relatively small cumulative transverse displacement and better deformation resistance.

BF1.25 (1.25% fiber content) showed the earliest positive deformation and the most rapid growth, consistent with its lowest flexural strength. This suggests that excessive fiber addition caused interfacial weakening and increased sensitivity to early transverse displacement. The positive deformation of BF0.5 (0.50% fiber content) increased steadily after 12 s and remained lower than that of BF0.75 and higher-content groups throughout the later loading stage, indicating that 0.50% fiber effectively constrained transverse opening at the crack location and delayed unstable deformation. The development of positive transverse deformation at the crack location showed a negative correlation with flexural strength: higher strength corresponded to smaller cumulative positive transverse deformation at the same loading stage. The 0.50% basalt fiber content therefore achieved the highest flexural strength while maintaining a favorable capacity to control transverse deformation.

### 3.5. DIC-Based Compressive Stress–Strain Analysis

**Figure 7:**
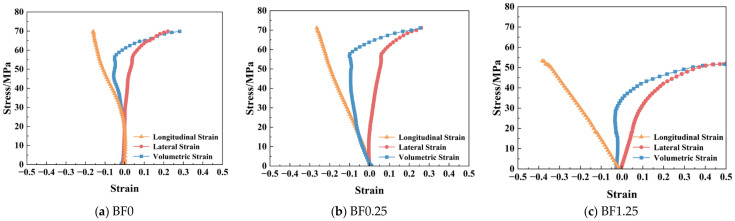
Stress–strain curves under compression.

Figure 7 shows the stress–strain curves of 28-day geopolymer mortar specimens BF0, BF0.25, and BF1.25 under uniaxial compression. The BF0 group exhibited relatively high peak stress and typical brittle failure behavior: after an initial linear-elastic segment, the curve entered a plastic-yielding stage, followed by a rapid stress drop after the peak. The transverse strain was negative in the early stage, indicating transverse contraction during initial compaction, and then became positive and gradually increased, reflecting the Poisson expansion effect. The BF0.25 group exhibited the highest peak stress and a markedly increased axial strain, indicating improved deformation capacity. Its transverse strain remained positive, without obvious initial compaction shrinkage, suggesting that fiber reinforcement improved material homogeneity. The BF1.25 group showed a substantially lower peak stress, but both axial and transverse strains increased considerably, indicating greatly enhanced ductility at the expense of strength. The elastic modulus was calculated from the initial linear segment of the stress-axial strain curve. The BF0 group had the highest elastic modulus and the lowest Poisson’s ratio. With increasing fiber content, the elastic modulus decreased, and Poisson’s ratio increased, indicating that fiber addition reduced material stiffness while enhancing transverse deformation capacity.

The volumetric strain of the BF0 group first developed in the compressive direction with increasing stress and then turned positive, with the transition point corresponding to crack initiation. In contrast, the volumetric strain of the BF0.25 group remained negative throughout the test, with no obvious volumetric expansion, indicating that the fibers effectively suppressed volume expansion caused by crack propagation. The volumetric strain of the BF1.25 group rapidly changed from negative to positive and showed pronounced volumetric expansion, consistent with highly ductile failure. The BF0 group exhibited brittle shear failure, characterized by slow axial strain development and a sharp increase in transverse strain at a later stage. The BF0.25 group exhibited a more ductile failure pattern; fiber bridging maintained specimen integrity, volumetric strain remained in the compressive range, and compressive strength was the highest. In the BF1.25 group, excess fibers introduced additional matrix defects, reducing strength, but substantially improved deformation capacity, producing plastic-flow-like behavior and significant volumetric expansion.

An appropriate basalt fiber content can optimize the compressive performance of geopolymer mortar by improving strength and suppressing volumetric expansion. Although an excessively high fiber content enhances ductility, it does so at the cost of strength. Therefore, fiber content should be balanced according to the target service conditions in practical applications. DIC observations effectively revealed how fibers regulate the evolution of the local strain field.

### 3.6. Microstructural Mechanism Analysis

#### 3.6.1. XRD

**Figure 8:**
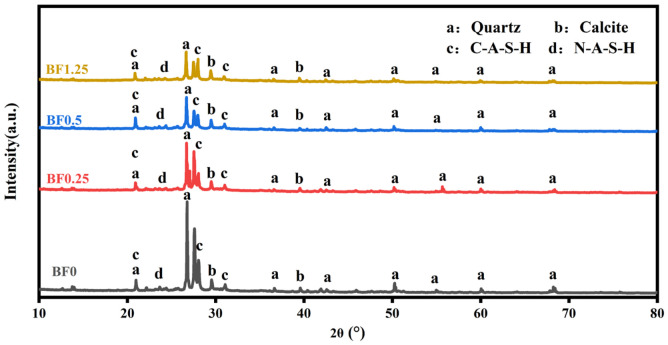
XRD patterns.

Figure 8 shows the XRD patterns of 28-day geopolymer mortar with different basalt fiber contents. All samples exhibited broad diffuse diffraction humps within 20–40° (2θ), corresponding to an amorphous aluminosilicate gel phase (N-A-S-H/C-A-S-H), indicating that basalt fiber addition did not alter the basic amorphous structure of the geopolymer. A small number of crystalline diffraction peaks were observed in all samples and were mainly assigned to unreacted gehlenite (Ca_2_Al_2_SiO_7_, PDF#00-035-0755), quartz (SiO_2_, PDF#00-046-1045), albite (Na(Si_3_Al)O_8_, PDF#00-010-0393), and the carbonation product calcite (CaCO_3_, PDF#01-083-0577) [38]. The peaks near 23.5° and 31.5° mainly corresponded to overlapping contributions from gehlenite and calcite, whereas the peaks near 38.0–38.5° were mainly attributed to gehlenite. No new crystalline phases associated with basalt fiber were detected, and no hydrotalcite phase was observed, which is consistent with the use of water glass as the alkali activator [53]. As fiber content increased, the intensity of the diffuse gel hump decreased slightly, presumably because the fibers acted as physical fillers and produced a weak dilution effect on the gel phase; however, the basic gel structure was not significantly affected. Because geopolymer gel is essentially amorphous, it appears as a diffuse hump in XRD patterns. During gel aging, locally ordered microcrystalline regions may form and be identified by phase-analysis software as crystalline minerals such as albite, thereby indirectly indicating the formation of a sodium-containing geopolymer gel (N-A-S-H).

#### 3.6.2. SEM

**Figure 9:**
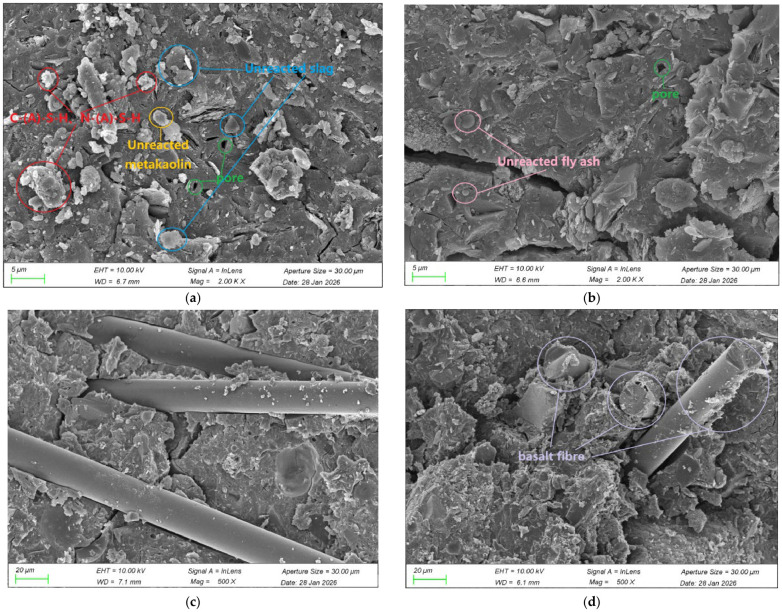
SEM micrographs: (**a**) and (**b**) are SEM images of the specimens without basalt fiber; (**c**) of the specimen with 0.5% basalt fiber; (**d**) of the specimen with 1.25% basalt fiber.

Figure 9 shows SEM micrographs of the metakaolin-slag-fly ash composite system at 28 days with different basalt fiber contents. The identification of different mineral and gel phases in this study was determined based on typical microscopic morphological characteristics reported in previous studies [54,55]. The matrix contained angular and irregular unreacted slag particles and flaky, poorly defined unreacted metakaolin particles, both of which were surrounded by continuous C-(A)-S-H and N-(A)-S-H gels. The gels showed a flocculent and dense morphology, although local pore defects remained visible. Unreacted fly ash microspheres were spherical and smooth; a small amount of gel adhered to some microsphere surfaces, indicating limited participation in the reaction [54]. The microstructural evolution with fiber content can be clearly observed. Images (a) and (b) correspond to the fiber-free specimen. Although the matrix was generally dense, the fracture surface was flat and smooth, showing typical brittle fracture morphology and a lack of effective energy-dissipation mechanisms. When 0.50% fiber was added (c), many traces of fiber pull-out or breakage were observed in the interfacial transition zone, and geopolymer gel adhered tightly to the fiber surface, indicating good fiber–matrix interfacial bonding. This combined effect of mechanical interlocking and chemical bonding provides the microstructural basis for the improved toughness of the composite. When fiber content increased to 1.25% (d), obvious fiber agglomeration appeared locally. Micropores and microcracks caused by fiber debonding were observed around agglomerates and within the interfacial transition zone, explaining the decrease in compressive strength at high fiber contents. Thus, an appropriate fiber content introduced a toughening mechanism without disrupting matrix continuity, whereas excessive fibers degraded the interfacial structure through agglomeration.

#### 3.6.3. FTIR

**Figure 10:**
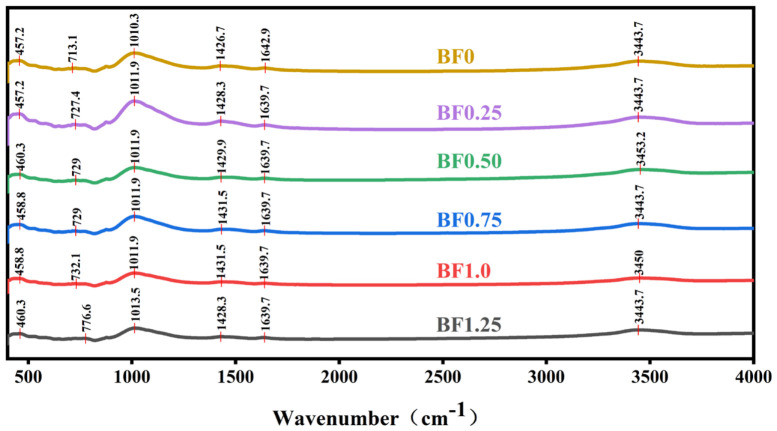
FTIR spectra.

Figure 10 shows the intensities of the main infrared absorption peaks of alkali-activated slag-fly ash-metakaolin geopolymer mortar with different basalt fiber contents. The characteristic peak intensities changed systematically with fiber content. The absorption band near 500 cm^−1^ is attributed to the bending vibration of Si-O-Si bonds. Its intensity remained stable (45.72–45.80) when fiber content ranged from 0% to 0.75%, but decreased markedly to 40.03 when the content increased to 1.00% and 1.25%, indicating that excessive fiber adversely affected the integrity of the silicate network [56]. The absorption band near 1000 cm^−1^ is characteristic of Si-O-T bonds in C-(N)-A-S-H gels. Its intensity followed a trend similar to that of the 500 cm^−1^ band: it remained stable at low fiber contents but weakened significantly at high contents, confirming that excessive fiber addition interfered with geopolymerization-product formation and reduced the degree of polymerization of the gel phase [57]. The absorption intensities in the ranges around 1500 cm^−1^ and 2000–4000 cm^−1^ also decreased in the high-fiber-content groups, further indicating reductions in structural water and total gel content [58,59]. Overall, FTIR analysis indicates that an appropriate basalt fiber content has no significant adverse effect on the geopolymer gel structure, whereas excessive fiber content results in a looser gel network and a lower degree of polymerization.

## 4. Conclusions

This study systematically investigated the effects of basalt fiber contents of 0%, 0.25%, 0.50%, 0.75%, 1.00%, and 1.25% on the workability, mechanical properties, and deformation behavior of a metakaolin-slag-fly ash ternary geopolymer mortar. Combined with 2D-DIC analysis and XRD, SEM, and FTIR characterization, the reinforcement and toughening mechanisms of basalt fibers were clarified. The main conclusions are as follows:

1. Basalt fiber incorporation significantly reduced geopolymer mortar flowability, and the reduction increased with fiber content. At 1.25% fiber content, flowability decreased by 21.0% compared with the fiber-free control, mainly because of the high specific surface area of the fibers and their physical cross-linking and entanglement within the paste. This trend is consistent with previous research on fiber-reinforced geopolymers [45].
2. Flexural strength first increased and then decreased with increasing fiber content. At 0.50% fiber content, the 3-day and 28-day flexural strengths reached 7.8 MPa and 12.6 MPa, respectively, representing increases of 20% and 8.2% relative to the control. Compressive strength increased slightly at low fiber contents at early ages; however, at 28 days, only the 0.25% group remained slightly higher than the control, and the compressive strength decreased continuously when the fiber content reached 0.50% or higher. Fibers play a crack-bridging role at early ages, while fiber agglomeration causes performance degradation at high dosages, which agrees with existing findings [50].
3. The 2D-DIC-based deformation analysis showed that fiber incorporation significantly increased the ultimate displacement of the specimens. The Y-direction ultimate displacements for the 0.50% and 0.75% groups were 125% and 113% higher, respectively, than those of the fiber-free group, demonstrating clear toughening effects. The transverse deformation evolution further showed that the 0.50% group entered a stable deformation stage earlier and exhibited moderate deformation growth, which was beneficial for delaying crack propagation. Appropriate fiber addition effectively improves the toughness and crack resistance of geopolymer materials [51].
4. Stress–strain analysis further confirmed that an appropriate fiber content (0.25%) improved compressive deformation capacity without significantly reducing strength and also suppressed volumetric expansion. Excessive fiber content (1.25%) greatly enhanced ductility but caused a substantial strength reduction and produced a plastic-flow-like failure mode. Excess fibers introduce internal defects and alter the failure characteristics of composites.
5. Microstructural analysis showed that basalt fiber addition did not change the amorphous gel structure of the geopolymer. At an appropriate content (0.50%), good interfacial bonding formed between fibers and the matrix, with a dense gel adhering to the fiber surface and providing bridging and crack-resisting effects. At excessive content (1.25%), fiber agglomeration introduced micropores and microcracks, degraded the interfacial structure, and weakened mechanical properties. Interfacial bonding state directly determines the macroscopic mechanical performance of fiber-reinforced geopolymers.

In summary, a basalt fiber content of 0.25–0.50% allowed the geopolymer mortar to maintain acceptable workability while achieving relatively high flexural strength and excellent deformation capacity; this range is therefore recommended as the optimal dosage. These results provide theoretical guidance and experimental support for the mix design and engineering application of high-performance, low-carbon geopolymer mortar.

## Figures and Tables

**Table 1:** Chemical composition of raw materials (%, provided by the suppliers).

Binder	SiO_2_	Al_2_O_3_	Fe_2_O_3_	CaO	MgO	SO_3_	Balance *
Metakaolin	52.22	43.49	0.63	0.52	1.09	0.37	1.68
Slag	31.44	14.29	0.31	42.07	8.64	2.18	2.07
Fly ash	69.35	18.52	4.1	5.02	2.14	0.08	0.79

* The balance was determined by difference from 100% using the major oxide composition provided by the supplier, which may cover minor components as well as loss on ignition (LOI).

**Table 2:** Parameters of the water glass solution (provided by the supplier).

Modulus	Baumé Degree	SiO_2_ (%)	Na_2_O (%)	H_2_O (%)
2.3	50	30	13.5	56.5

**Table 3:** Properties of basalt fiber (provided by the supplier).

Fiber	Density (g/cm^3^)	Material	Dia. (μm)	Strength (MPa)	Modulus (GPa)	Length (mm)	Elong. (%)	Alkali Res. (%)
Basalt fiber	2.62	Basalt ore	17	1938	76.1	6	2.9	75

**Table 4:** Mix proportions of geopolymer mortar.

Specimen No.	Water (g)	Metakaolin (g)	Slag (g)	Fly Ash (g)	Sand (g)	Alkali Activator (g)		Basalt Fiber	
						NaOH	Water Glass	Mass Content (%)	Mass (g)
BF0	103.95	202.5	202.5	45	1350	16.72	174.42	0	0
BF0.25	103.95	202.5	202.5	45	1350	16.72	174.42	0.25	5.24
BF0.5	103.95	202.5	202.5	45	1350	16.72	174.42	0.50	10.48
BF0.75	103.95	202.5	202.5	45	1350	16.72	174.42	0.75	15.71
BF1.0	103.95	202.5	202.5	45	1350	16.72	174.42	1.00	20.95
BF1.25	103.95	202.5	202.5	45	1350	16.72	174.42	1.25	26.19

## Data Availability

The original contributions presented in this study are included in the article. Further inquiries can be directed to the corresponding author.
